# Nacre-Mimetic Green Flame Retardant: Ultra-High Nanofiller Content, Thin Nanocomposite as an Effective Flame Retardant

**DOI:** 10.3390/polym12102351

**Published:** 2020-10-14

**Authors:** Irlaine Machado, Isabel Hsieh, Veronica Calado, Thomas Chapin, Hatsuo Ishida

**Affiliations:** 1 Department of Macromolecular Science and Engineering, Case Western Reserve University, 10900 Euclid Avenue, Cleveland, OH 44106-7202, USA; ixm121@case.edu; 2 Hathaway Brown School, Shaker Heights, OH 44122, USA; ihsieh21@hb.edu; 3 School of Chemistry, Universidade Federal do Rio de Janeiro, Rua Horácio Macedo 2030, Cidade Universitária, Rio de Janeiro, RJ 21941-909, Brazil; calado@eq.ufrj.br; 4 Underwriters Laboratories Inc. (UL), 2500 Dundee Rd., Northbrook, IL 60062S, USA; J.Thomas.Chapin@ul.org

**Keywords:** benzoxazine, polybenzoxazine, green flame retardant, polyurethane foam

## Abstract

A nacre-mimetic brick-and-mortar structure was used to develop a new flame-retardant technology. A second biomimetic approach was utilized to develop a non-flammable elastomeric benzoxazine for use as a polymer matrix that effectively adheres to the hydrophilic laponite nanofiller. A combination of laponite and benzoxazine is used to apply an ultra-high nanofiller content, thin nanocomposite coating on a polyurethane foam. The technology used is made environmentally friendly by eliminating the need to add any undesirable flame retardants, such as phosphorus additives or halogenated compounds. The very-thin coating on the polyurethane foam (PUF) is obtained through a single dip-coating. The structure of the polymer has been confirmed by proton nuclear magnetic resonance spectroscopy (^1^H NMR) and Fourier transform infrared spectroscopy (FTIR). The flammability of the polymer and nanocomposite was evaluated by heat release capacity using microscale combustion calorimetry (MCC). A material with heat release capacity (HRC) lower than 100 J/Kg is considered non-ignitable. The nanocomposite developed exhibits HRC of 22 J/Kg, which is well within the classification of a non-ignitable material. The cone calorimeter test was also used to investigate the flame retardancy of the nanocomposite’s thin film on polyurethane foam. This test confirms that the second peak of the heat release rate (HRR) decreased 62% or completely disappeared for the coated PUF with different loadings. Compression tests show an increase in the modulus of the PUF by 88% for the 4 wt% coating concentration. Upon repeated modulus tests, the rigidity decreases, approaching the modulus of the uncoated PUF. However, the effect of this repeated mechanical loading does not significantly affect the flame retarding performance.

## 1. Introduction

Flame retardants (FRs) are chemicals added to materials to protect them against fire. This is achieved by preventing the combustion of material, reducing the amount of combustible fragments emitted, isolating the combustible fragments from oxygen, and/or reducing the heat generated that further decomposes the polymer [1,2,3].

The most common flame retardants used for polymers are halogenated elements, phosphorous, nitrogen, and inorganic compounds, such as boron, alumina, and magnesium hydroxide. However, Some of the most widely used flame retardant technologies such as halogenated flame retardants (HFR) and phosphorous flame retardants (PFRs) are prohibited or discouraged for use due to their toxic combustion products [4,5]. Environmental issues caused by traditional flame retardants have led to an increasing demand for new greener flame retardation methods for polymers. New studies are taking much greener approaches. These include polymer nanocomposites, intumescent systems [6], char formers, additives found in natural renewable resources [7], and polymer morphology modification [3].

Polyurethane foam (PUF) is an important class of polymer that has a wide application on residential furniture and mattresses. However, these comfortable items can be quite hazardous. They are responsible for most of the fatalities and injuries in residential fires [8]. A newer, environmentally friendly flame retardant approach that has called attention is a flame retardant nanocoating prepared using a layer-by-layer (L-B-L) method. This technology seems to be effective in decreasing the severity of the PUF flammability and other substrates. The layer-by-layer (LBL) technique is used to fabricate nanocomposite thin films and achieve a nanoscale uniformity. The LBL approach is achieved through the sequential adsorption of nanoscale layers of oppositely charged components, such as polymer and inorganic particles. After each layer film is adsorbed onto the substrate, it is washed before the adsorption of the different layer film [9]. Grunlan and coworkers [10] assembled a multilayer intumescent coating using renewable polyelectrolytes, such as cationic chitosan and anionic phytic acid, into 30 bilayers. Chitosan and phytic acid combined can provide an intumescent system. They used cotton fabric as a substrate that acts as a carbon source. The intumescent system was deposited on the substrate via layer-by-layer assembly causing 60% reduction in the peak heat release rate (pHRR) and a 76% decrease in total heat release (THR), as determined by microcombustion calorimetry (MCC).

Davis and collaborators [11] fabricated a layer-by-layer nanocomposite coating using anionic poly (acrylic acid) monolayer between anionic clay and polyethylenimine (PEI) monolayers. The eight trilayer nanocomposite structure was deposited on PUF, reducing its flammability to 17% pHRR and 21% of the total burn time. Both studies used a greener solution and demonstrated reduction in flammability that was comparable to common commercial flame retardants. Different studies that applied flame retardant nanocoating prepared by LBL on textile, foams, and bulk polymers showed a significant reduction in heat release rate and smoke release [12]. Those results are very promising for the future of environmentally friendly flame retardants.

The problem becomes that sequential immersion and washes of the substrate into the solutions are necessary to prepare these multilayer films with good properties, making it unattractive when applied on a large scale due to the laborious multistep procedure [13]. In this context, researchers have been taking lessons from nature to manufacture materials with higher properties. Due to their well-organized hierarchical structure, natural materials can create nanocomposites with a high content of inorganic components and achieve a combination of excellent strength, stiffness, and high toughness. These materials include bone (~40 vol%), sponge spicule (89 vol%), nacre (95 vol%) [14,15], and bamboo (80 wt%) [16]. Nacre, also known as mother-of-pearl, consists of 95 vol% polygonal platelet-shaped aragonite (CaCO_3_) crystals of single platelet with a diameter of 5–8 μm. This natural material has approximately 200–900 nm thickness incorporated into a 5 vol% of a biopolymer matrix of about 10–50 nm thickness. It consists of an insoluble chitin sheet between a layer of soluble proteins, and shows a brick-and-mortar structure, leading to a synergistic mechanical performance [13,15,17,18,19] and exceptional stability [20,21,22].

Natural nanocomposites can load-bear much higher content of nanofiller than many traditional man-made nanocomposites reported in the literature. Nacre provides an excellent guideline to produce a nanocomposite with a high content of nanofiller as it overcomes the problems of poor dispersion, low loading, and weak interfacial interactions while improving the fire resistance of a material [17,18,19,20,21].

Here, we report a single dip-coating self-assembly approach to prepare a very thin, bioinspired film in a brick-and-mortar structure on PUF. Inspired by the structure and properties of the nacre, the nanocomposite thin film will be prepared by developing a biomimetic composite structure of the nacre. In order to obtain the structure, an elastomeric benzoxazine that will have good compatibility with a hydrophilic surface of laponite will be synthesized as a matrix with high nanofiller content of laponite.

It is important to clarify the difference of the nanocomposite the current paper attempts to prepare from the traditional clay-based nanocomposite many papers have reported. Laponite, a synthetic trioctahedral clay mineral whose structural formula is Na_0.7_Si_8_Mg_5.5_Li_0.3_O_20_(OH)_4_ [23,24] clay, has layers of silicate that are stacked to make a coin-like appearance with typical diameter of about 25 nm [13]. As the definition of nanocomposite is a composite material with at least one dimension is on the order of nanometers, typically less than 100 nm, even without pealing each silicate layer in an exfoliated manner, a laponite-filled polybenzoxazine can be considered a nanocomposite. Thus, the laponite composites with or without exfoliation of each silicate layer can both be considered nanocomposites with expected differences in processability. It is this latter type of nanocomposite the current paper attempts to prepare since it presents much less processing difficulties, as long as such form of nanocomposite can achieve a desired nacre-like morphology.

Polybenzoxazine is a class of polymer that has attracted attention due to its excellent properties, such as near-zero shrinkage upon polymerization, high char yield, the fast development of the mechanical and physical properties with the degree of conversion, and high design flexibility at the molecular level [25,26]. One of the properties that is drawing attention to this polymer is its extraordinary flame retardant property [27,28,29,30,31]. Benzoxazine-based polymers have excellent flame-retardant properties owing to the phenolic and tertiary amine groups inside its structure, which are considered anti-flammable. According to Walters and Lyon, the molar group contribution of a phenolic and tertiary amine, both of which exist in polybenzoxazine, is significantly more effective than other typical group units [32].

Most of the published articles related to benzoxazine flame-retardant use a bisphenol-A based benzoxazine, which does not have a good limiting oxygen index (LOI) [33,34]. It is well known that halogenated flame retardants produce toxic gases upon incineration that are not environmentally safe [35]. As an alternative, flame retardants containing phosphorus [35,36] have been used to reduce the high combustibility of polymer materials. The same phosphorus approach is also effective in improving the flame retardancy of polybenzoxazin [34,37,38]. However, there is some concern for phosphorous compounds from a toxicity point of view [5]. Thus, there is a need for an environmentally friendly, flame-retardant material free of phosphorus and halogenated compounds. Such a polymer with good flame-retardant behavior is desired for building construction, furniture, electrical applications, electronics, such as appliances, wire, and cables, and transportation, such as automobile, aviation, and aerospace [35,36].

This project aims to develop a new flame-retardant technology that uses an elastomeric polybenzoxazine with excellent flame retardation in combination with an ultra-high nanofiller content nanocomposite to be applied as a simple, single-dip coating. By using a high content of nanofiller and a flexible polymer to obtain a nanocomposite, a unique morphology mimicking the brick-and-mortar structure of nacre can be obtained which effectively blocks the diffusion of fuel from a burning substrate and oxygen from the atmosphere reaching the burning surface.

A biomimetic technology is also applied to the synthesis of benzoxazine. Catechol is used as the phenolic component of benzoxazine in order to mimic the remarkable adhesive property of a marine mussel, especially to a hydrophilic surface even in the presence of water. The primary amine selected is aminopropyl terminated polydimethylsiloxane (PDMS) that contains an elastomeric segment in its structure. Furfurylamine, a natural renewable amine, is chosen as a chain end to act as a cross-linkable group [39,40] to further enhance the cross-linking ability of benzoxazine. By using difunctional phenol and difunctional amine, a main-chain type benzoxazine polymeric precursor is synthesized, incorporating the benzoxazine unit as the chemical repeat unit. The cross-linked polybenzoxazine derived from the main-chain type benzoxazine, which is abbreviated as CA-pdms-fu, might possess an excellent combination of properties such as high thermal stability, low flammability, excellent adhesion, and flexibility. This main-chain type oligomer possesses cross-linkable groups as part of each chemical repeat unit. Polybenzoxazines typically possess three degradation temperature regimes where the lowest temperature around 300 °C is due to the cleavage of the chain ends or dangling side chains, 400–500 °C due to the evaporation of the amine moieties, and finally around 500–600 °C due to the phenolic structures [41]. It has been reported that the main-chain type polybenzoxazine either significantly reduce or eliminate the lowest degradation temperature by minimizing the formation of the dangling chain ends in the cross-linked structure [42]. The polybenzoxazines derived from the main-chain type polybenzoxazine precursors are more ductile than those derived from monomeric precursors, again due to the reduction of the molecular defects caused by the chain ends [43]. The flexible polydimethylsiloxane chain is added to further enhance the ductility of the already improved main-chain type polybenzoxazine. It is important to emphasize that this main-chain polybenzoxazine flame retardant does not make use of halogenated or phosphorous compounds. In addition to the elimination of these undesirable components, this compound also contains two components, catechol and furfurylamine, derived from natural renewable resources, making it a further green, environmentally flame retardant.

Considering this, a green flame-retardant nanocomposite with high nanofiller content has been made using a non-flammable elastomeric polybenzoxazine as a matrix. This unique green flame-retardant nanocomposite is applied as a very thin nanocomposite coating on polyurethane foam (PUF). As a result of the elastomeric polybenzoxazine matrix, it is expected that the influence of the ultra-thin coating on the mechanical properties of PUF will be minimized.

## 2. Experimental

### 2.1. Materials

Aminopropyl-terminated polydimethylsiloxane (molar mass molecular weight = 850 g/mol) was purchased from Gelest, Morrisville, PA, USA. Catechol (≤99%) and paraformaldehyde (≤96%) were purchased from Aldrich Chemical Company, St. Louis, MO, USA. Toluene was obtained from Fisher Scientific Company, Hampton, NH, USA and ethanol was obtained from Decon Laboratories Inc., King of Prussia, PA, USA. Laponite^®^ RD (LAP) (registered trademark of the company BYK A dditives Ltd.) was provided by BYK USA Inc., Gonzales, TX, USA. Untreated polyurethane (PU) foam was kindly supplied by Underwriters Laboratory, Columbia, MD, USA.

### 2.2. Characterization

^1^H nuclear magnetic resonance (^1^H NMR) spectra were recorded on a Varian Oxford A600 at a proton frequency of 600 MHz in CDCl_3_. The average number of transients used for ^1^H measurement was 32. The relaxation time of 10 s was used for the integrated intensity determination of the ^1^H NMR spectra. Fourier transform infrared spectroscopic (FTIR) spectra of the benzoxazine monomers and polymers were obtained on an ABB MB3000 FTIR spectrometer, which is equipped with a deuterated triglycine sulfate (DTGS) detector and a dry air purge unit. Coaddition of 16 scans was recorded at a resolution of 4 cm^−1^ in the frequency range of 4000–400 cm^−1^. A TA Instruments differential scanning calorimeter (DSC) model 2920 was used with a temperature ramp rate of 10 °C min^−1^ and a nitrogen flow rate of 60 mL min^−1^ for all tests of DSC study. Thermogravimetric analysis (TGA) was conducted on a TA Instruments model 2950 TGA with a heating rate of 10 °C/min under nitrogen and air at a flow rate of 60 mL/min unless otherwise indicated. For determining the activation energies of thermal decomposition, various scanning rates at 5, 10, 15, 20, and 25 °C/min were used. Scanning electron microscopy (SEM) and energy-dispersive X-ray spectroscopy (EDX) was conducted with a FEI Helios 650 field emission scanning electron microscope with focused ion beam with EDX. SEM samples were sputter-coated with a thin layer of conducting material before testing. Compression testing was performed by an MTS machine (Model 5565) with a compression testing fixture at a cross-head speed of 10 mm/min using 1 kN load cell to measure the applied force in a coated polyurethane foam cut into 4 cm cubes. Mechanical properties were evaluated using an MTS Insight (Electromechanical–kN Standard Length) testing machine. Tensile tests were conducted at ambient temperature on at least five specimens, using cross-head speeds of 15 mm/min. Flammability results of the polymers and coating were obtained with a microscale combustion calorimeter (MCC) originally developed by Federal Aviation Administration (FAA). The MCC was manufactured by Fire Testing Technology, East Grinstead, UK. The samples were heated to 800 °C at a heating rate of 1 °C/min under nitrogen. The nitrogen flow in the pyrolysis chamber was 80 mL/min. The flow rate of oxygen in the combustor chamber was 20 mL/min. The samples were run in triplicate. Coated PUF flammability was evaluated with a cone calorimeter manufactured by Fire Testing Technology, UK, with ASTM E 1354 standard. PUF samples were prepared with 10 cm × 10 cm × 4.0 cm dimensions and were tested under heat flux of 35 kW/m^2^ with the exhaust flow rate of 24 L/s. All PUF specimens were wrapped with heavy-duty aluminum foil with exposed top surface to heat.

### 2.3. Preparation of Main-Chain Benzoxazine Oligomers 8,8′-((1,1,3,3,5,5-Hexamethyltrisiloxane-1,5-Diyl) bis(Propane-3,1-Diyl))bis(3-(Furan-2-Ylmethyl)-2,3,4,7,8,9-Hexahydrobenzo [1 –e:4,3-e’]bis([1,3]Oxazine)), (Abbreviated as CA-Pdms-Fu)

**Scheme 1:**
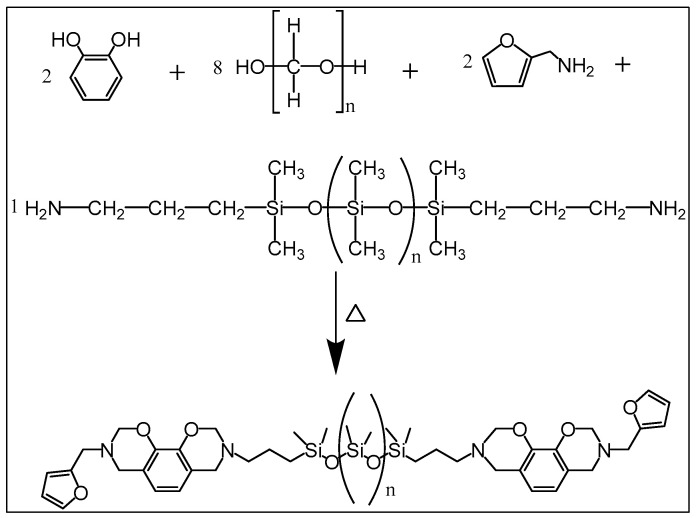
Synthesis of CA-pdms-fu.

Scheme 1 shows the synthetic routes used to successfully incorporate the reactants into the same molecular structure to obtain the desired benzoxazine based on the following synthetic procedures. A one-pot Mannich condensation reaction was used to obtain the benzoxazine. The oligomer was prepared in a 25 mL round-bottom flask. A mixture of catechol (20 mmol, 2.202 g), paraformaldehyde (80 mmol, 2.402 g), aminopropyl-terminated poly (dimethylsiloxane) (10 mmol, 8.75 g) and furfurylamine (20 mmol, 1.942 g) in 75 mL of toluene were mechanically stirred at 90 °C for 5 h. The crude product was washed three times with distilled water. Then, the solution was dried over anhydrous magnesium sulfate (MgSO_4_) and filtered. The solvent was removed under vacuum affording a viscous brown resin (Yield: 75%).

### 2.4. Preparation of Benzoxazine Oligomer Films

The films were prepared using a casting method. A solution of 30 wt% of the synthesized main-chain type polybenzoxazine oligomers was dissolved in toluene. Then, the film was cast over Teflon plates. The film was first dried in a chamber saturated with toluene for homogeneous evaporation of the solvent. Then, the film was dried in a vacuum oven at 60 °C for 24 h to remove the solvent. The film was transparent with a thickness of 0.8 mm.

### 2.5. Preparation of Elastomeric Benzoxazine/Laponite Nanocomposite

The nanocomposite coating was prepared using the elastomeric polybenzoxazine synthesized, and laponite. A solution of 1 wt% of CA-pdms-fu was prepared by dissolving the monomer in ethanol and mechanically stirring for 24 h. A laponite suspension was prepared by adding laponite to deionized water and vigorously stirring for 24 h. The resulting solution of CA-pdms-fu was added over the Laponite suspension (1 wt%), in the amounts corresponding to the concentrations of 10 wt% and 90 wt% of CA-pdms-fu and Laponite, respectively. to the 1 wt% laponite suspension to obtain a laponite filler content of 90 wt% with respect to the total solid content upon evaporation of the solvent. The prepared nanocomposite was cast on a petri dish and dried.

### 2.6. Single Dip Coating of Very-Thin Film Nanocomposite on Polyurethane Foam

A Polyurethane foam sample with dimensions of 10 cm × 10 cm × 4.0 cm was dipped into a suspension of laponite in the main-chain type polybenzoxazine oligomer solution as described above. It was submerged and squeezed until no more bubbles formed. Then, the excess amount of mixture was squeezed out with a constant force. The foam was dried in a vacuum oven at 100 °C for 1 h, and it was kept overnight at 80 °C to remove water and ethanol. After drying, the polyurethane foam coated with the nanocomposite was placed into a desiccator. The 1 wt% polybenzoxazine/laponite suspension (90:10 wt% ratio of the laponite and benzoxazine matrix) was used to coat polyurethane foam with different uptake of 2, 3, 4, 5, 6, 10, and 18 wt%.

Coated polyurethane foam with different uptakes was achieved applying a constant force using steel plates of different weights on the soaked PUF. Each sample was squeezed a specific number of times to obtain the desired adsorption.

## 3. Results and Discussion

In an attempt to understand the effect of a very thin coating on PUF made by ultra-high nanofiller content nanocomposite using a strategically synthesized benzoxazine to perform as an elastomeric matrix, structural, thermal, mechanical and flammability tests were performed. One of the challenges of this study is to synthesize a benzoxazine that exhibits both elastomeric and flame-retardant characteristics since the flammability of elastomers is usually high.

**Figure 1:**
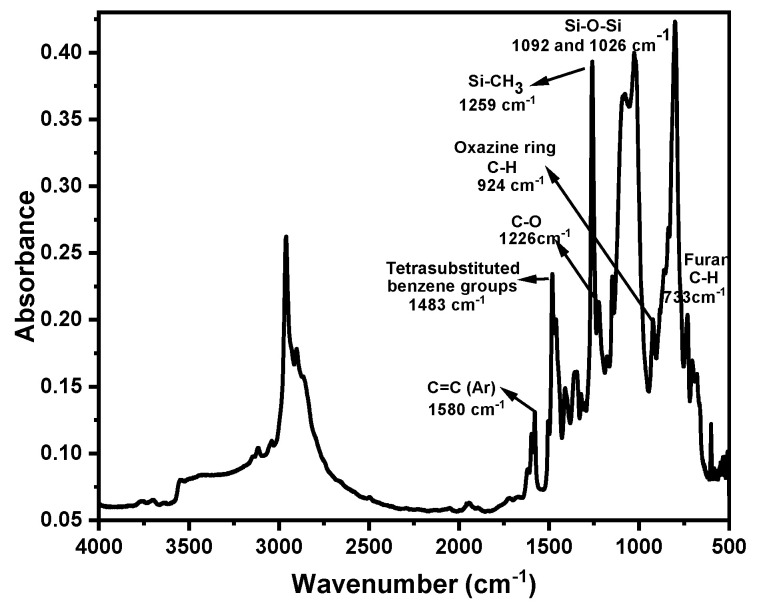
FTIR spectrum of CA-pdms-fu.

The chemical structure of benzoxazine oligomers was characterized by FT-IR, and ^1^H NMR. Figure 1 shows the FTIR spectra of CA-pdms-fu monomers and the polymer derived therefrom. The spectrum of benzoxazine monomers is consistent with its structure. The characteristic absorptions of the benzoxazine ring structure appeared at 1226 cm^−1^ due to the asymmetric modes of the C–O–C. The C–N–C symmetric stretching vibrations occurred at 1150 cm^−1^. The characteristic oxazine ring mode appeared at 924 cm^−1^ [44]. The incorporation of furan into the molecular structure is confirmed by the presence of the vibrations at 1503, 1062 overlapped, and 733 cm^−1^ which are assigned to the C=C stretching, C–O asymmetric stretching, and C–H out-of-plane in phase wagging of the furan ring, respectively. The strong band at 1092 and 1026 cm^−1^ are assigned to the Si–O–Si stretching band of the siloxane units. The well-known Si–CH_3_ deformation of PDMS appeared at 1259 cm^−1^. The band at 1580 cm^−1^ corresponds to the C=C stretching vibration, and the vibration around 1483 cm^−1^ is assigned to the in-plane CH deformation of tetrasubstituted benzene ring. The absorption bands discussed above are all in agreement with the structure of the designed compound.

**Figure 2:**
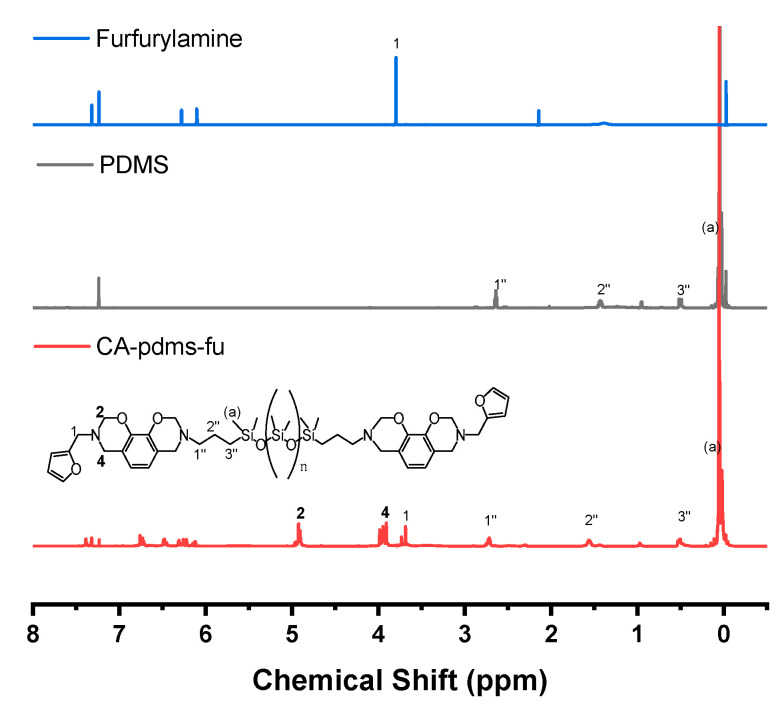
^1^H NMR spectra of CA-pdms-fu in CDCl_3_ and its reactants.

The ^1^H NMR spectra, shown in Figure 2, further confirmed the structure of CA-pdms-fu and its reactants. The resonance of Si–CH_3_ protons appeared at 0.0–0.01 ppm. The resonances of Si–CH_2_–CH_2_, Si–CH_2_–CH_2_, and N–CH_2_ protons appeared at 0.5, 1.5, and 2.7 ppm, respectively, while the protons of CH_2_ of the furfurylamine appeared at 3.7 ppm. In our main-chain type benzoxazine, they appeared in the range 4.90–4.92 ppm for the O–CH_2_–N group and 3.91–3.99 ppm for the Ar–CH_2_–N group. Due to the possibility of different magnetic environments, multiple resonances for the CH_2_ group are possible and a very detailed study is needed before we can assign each resonance. Nonetheless, they are in the general range of the characteristic resonances of the oxazine ring that are 0.6–0.9 ppm apart [45].

**Scheme 2:**
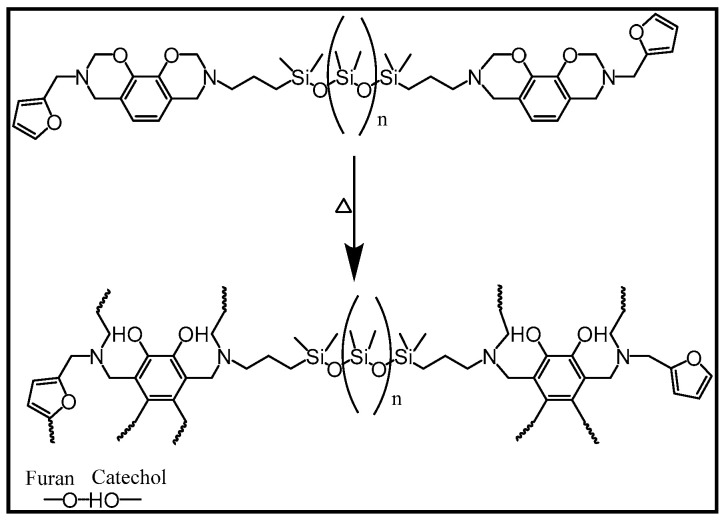
Polymerization of poly(CA-pdms-fu).

**Figure 3:**
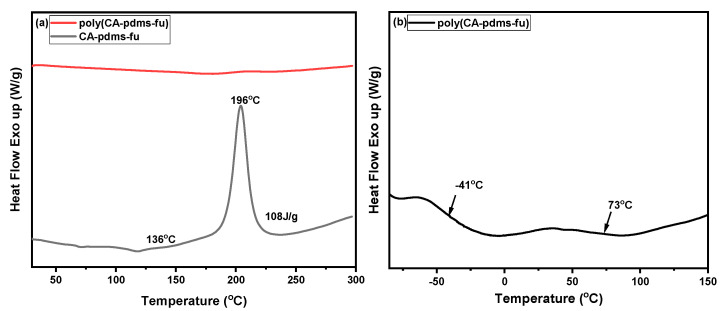
DSC thermograms for (**a**) CA-pdms-fu, lower curve and poly(CA-pdms-fu), upper curve and (**b**) DSC thermogram showing glass transition temperature of poly(CA-pdms-fu).

The polymerization behavior of poly(CA-pdms-fu) in Scheme 2 was studied by differential scanning calorimeter (DSC). The results are shown in Figure 3.

**Figure 4:**
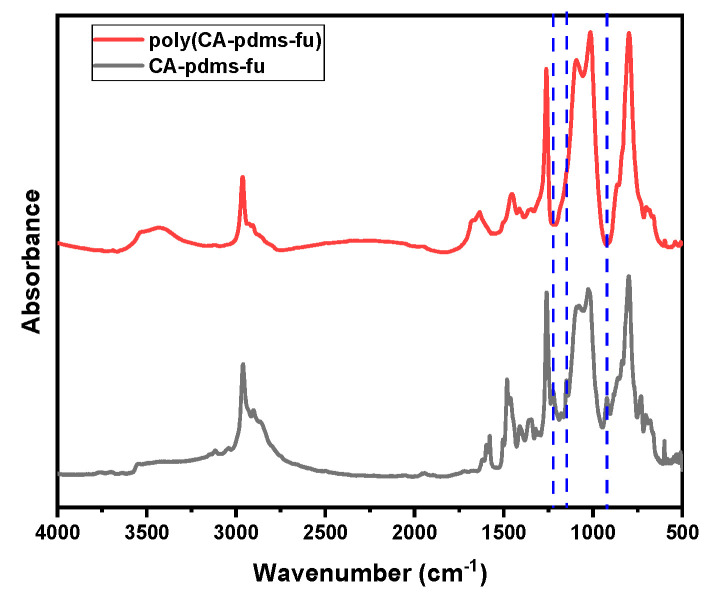
FTIR spectra of CA-pdms-fu and poly(CA-pdms-fu).

As can be seen in Figure 3, the DSC thermogram of CA-pdms-fu after drying at 60 °C for 24 h showed a single exothermic peak, which corresponds to the ring opening polymerization of the oxazine. This peak is typically used as an indicator of the polymerization temperature. The onset temperature for CA-pdms-fu is 136 °C, and the maximum temperature is 196 °C, which is significantly lower than the conventional benzoxazine BA-a (bisphenol-A and aniline based benzoxazine monomer) that exhibits polymerization exothermic temperature of 260 °C. The FTIR spectrum of CA-pdms-fu shows a small amount of phenolic OH groups in the range of 3200–2800 cm^−1^ due to the chelated OH groups. The existence of ring-opened structures can contribute to lower the polymerization temperature [46,47]. However, the ^1^H NMR spectrum indicates relatively high purity. Thus, this low polymerization exothermic is not just due to the residual OH groups, but is likely due to the intrinsic property of the material. Typically, electron-withdrawing and acidic phenol species provide a strong catalytic effect, which reduces the ring-opening polymerization temperature [48,49]. However, the multi hydroxyl group benzene ring as OH is not an electron-withdrawing group; it is a very strong electron-donating. In this case, what accelerates the ring-opening polymerization is the multiple hydroxyl groups, which lower the polymerization temperature. Hydroxyl groups accelerate the polymerization by forming a hydrogen bond between a hydroxyl group and oxazine ring, which catalyzes the ring-opening polymerization [50,51]. The DSC thermogram in Figure 3a shows the poly(CA-pdms-fu) after the polymerization at 146 °C for 2 h in a convection oven. The second run of the DSC analysis did not show an exothermic peak in the range from 146 °C to 250 °C, confirming the polymerization of oxazine rings. To further confirm this statement, the FTIR spectrum of poly(CA-pdms-fu) was obtained as shown in Figure 4. It was noticed that the characteristic bands of benzoxazine ring at 1226 cm^−1^ assigned as asymmetric vibrations of C–O–C, and the vibration at 1150 cm^−1^ of C–N–C disappeared after the cross-linking process. Additionally, the band at 924 cm^−1^ of the benzoxazine ring [45] disappeared, indicating that the oxazine ring opened that proceeds the polymerization. A new very broad band in the range of 3500–3300 cm^−1^ is developed, which is assigned to the phenolic hydroxyl structure. Based on the above results from DSC and FTIR analyses, it can be concluded that the cross-linking reaction has taken place.

Glass transition temperature (*T*_g_) of the cross-linked polymer as shown in Figure 3b was observed at −41 °C. The low *T*_g_ is due to the incorporation of PDMS, which is a flexible component with *T*_g_ of −127 °C. There seems to be another transition, though it is less pronounced, at 73 °C. The catechol-based benzoxazine without PDMS component is expected to show a high *T*_g_ above 150 °C. When two immiscible c-omponents are copolymerized and forced to improve the compatibility without having intrinsic molecular miscibility, the two corresponding *T*_g_’s of the homopolymers tend to show shifts towards each other. Having observed up-shift of the PDMS *T*_g_ by Δ*T* = 86 °C, it is reasonable to suspect that this 73 °C transition is the benzoxazine portion of the cross-linked chains down-shifted from a higher temperature.

PDMS consists of an inorganic siloxane backbone that is uniquely flexible; this characteristic is related to its freedom of rotation about linkage due to the large Si–O–Si bond angle and its ability to adopt different conformations which confer to this polymer a low *T*_g_ [52].

**Figure 5:**
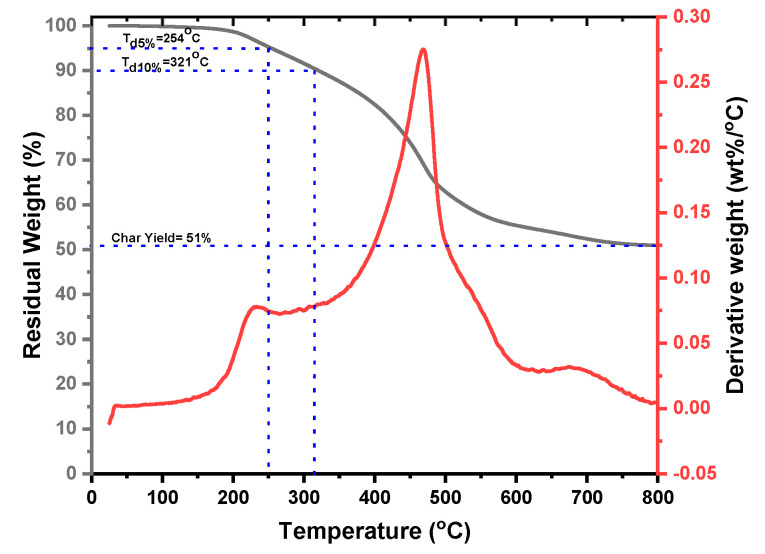
TGA (black curve) and DTG (red curve) thermogram of poly(CA-pdms-fu).

To investigate the thermal stability of the elastomeric, poly(CA-pdms-fu), thermogravimetric analysis (TGA) was performed. The thermogram is shown in Figure 5. Polybenzoxazine is known to present three-step decomposition stages that correspond to the breakage of chain end or branched amine, degradation of the Mannich base in the main chain, and evaporation of phenolic moieties [41,53]. In Figure 5, it is possible to see these steps and analyze the thermal stability of the sample. In the first region, the early weight loss at 228 °C might be related to the methyl scission group attached to the silicon [54], and also the alkylamine [55]. The second degradation stage at 469 °C, and the third peak, appears as a weak shoulder at 555 °C and the last weight loss at 694 °C that might be the crosslinked units generated. Usually, the second peak that is due to the evaporation of the amine moiety is stronger in the case of ordinary polybenzoxazines where a monofunctional primary amine is used. However, the structure of poly(CA-pdms-fu) is different in that the amine group also participate in the cross-linked network as the furane ring is reported to form bonds with neighboring groups and is no longer a dangling side chain [40,56]. This adds improved thermal stability which results in the significant reduction of the intensity of the second degradation peak. The 5% (*T*_d5_) and 10% (*T*_d10_) weight loss temperatures, were observed at 254 °C and 321 °C, respectively. The chair yield of the poly (CA-pdms-fu) is 51%, which is remarkably high for an elastomeric material. This is favorably compared with the char yield of about 51% at 800 °C of aniline polyphosphazene, which is considered to be a highly thermally stable and flame resistant elastomeric polymer [57].

**Figure 6:**
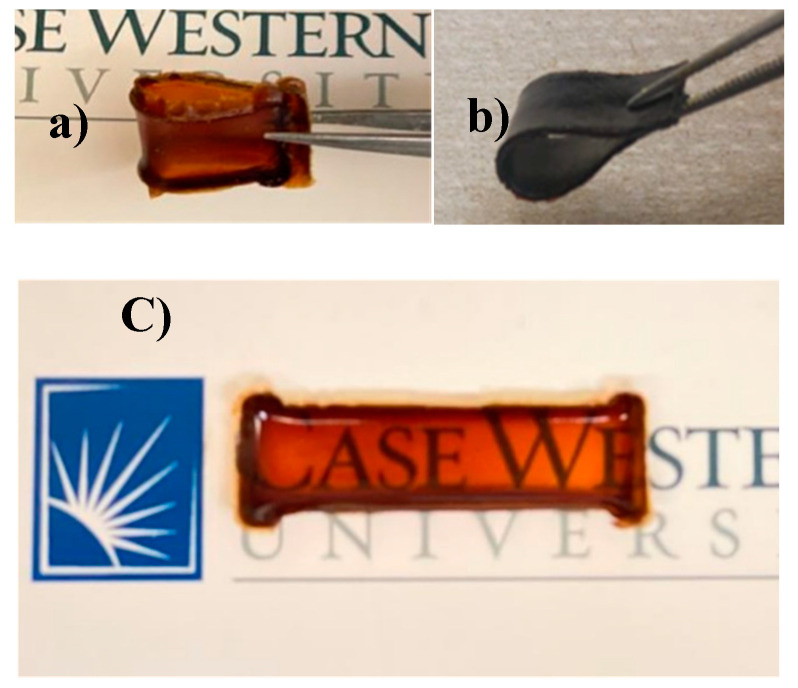
Optical photographs of CA-pdms-fu films (**a**,**b**) and poly (CA-pdms-fu) (**c**).

**Figure 7:**
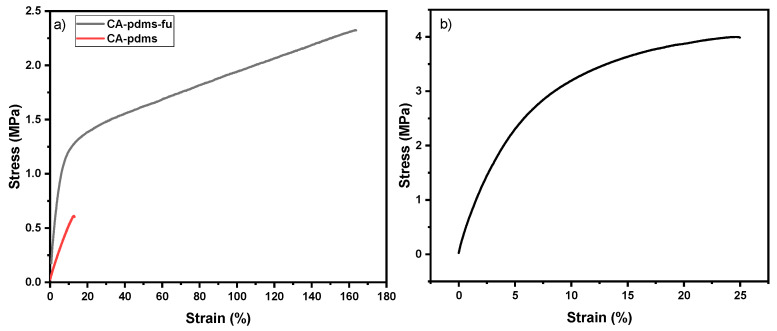
Stress-strain curve of (**a**) CA-pdms (red curve), and CA-pdms-fu (black curve), and (**b**) poly(CA-pdms-fu) films.

The tensile property measurements of ordinary polybenzoxazine presents something of a challenge due to its brittleness. However, it is rather straightforward for the mechanical property measurement of the current polybenzoxazine as it is quite flexible, as shown in Figure 6 and Figure 7.

The tensile test was performed to measure the elastic modulus, tensile strength, and elongation at the break that are summarized in Table 1. The most common polybenzoxazine synthesized from (bisphenol A, aniline), poly(BA-a), show poor toughness, which makes the film break at a low elongation of 1.6% [47]. The stress-strain curve of poly(CA-pdms-fu) suggests that the incorporation of polydimethylsiloxane (PDMS) in the main chain of polybenzoxazine significantly increases the elongation to break in comparison to poly(BA-a). However, even more striking is the tremendous increase in toughness simply by adding the furfurylamine terminal groups. The strain to break of the poly(CA-pdms-fu) is 25% which is much higher than the typical polybenzoxazine and other polybenzoxazine containing PDMS as a toughening agent [47,58,59] as can be seen in Table 1. It is surprising to see that poly(BA-a) that is copolymerized with flexible PDMS unit without furfurylamine terminal groups do not show a dramatic increase in elongation at break. It is thus hypothesized that the low toughness of polybenzoxazines, even when they are copolymerized with PDMS units, is due to the loose chain ends that act as molecular defects.

The elongation at break results are due to the low glass transition temperature of PDMS, which acts as the toughener. Together with the low elastic modulus value, it suggests that the synthesis of an elastomeric polybenzoxazine was successfully achieved, resulting in a low stiffness polymer.

To determine the kinetic parameters of thermal degradation, the Flynn-Wall-Ozawa method was applied [61]. This is an isoconversional method which is independent of the conversion function. From this method, it is possible to quantify the activation energy (E_a_) that can be obtained from the slope of the straight line by plotting log_10_β as a function of 1000/*T*_d_ as long as a single degradation mechanism is observed [62]. The Flynn-Wall-Ozawa method can be expressed by the Equation (1):

(1) $$\log_{10}\beta ={\;\log}_{10}\left(\frac{{\mathrm{AE}}_{a}}{R\left(\alpha \right)}\right)-2.315-0.4567\frac{E_{a}}{\mathrm{RT}}$$

where β is the constant heating rate, and T is the degradation temperature value at a given conversion α. E_a_ is the activation energy for thermal degradation, R is the gas constant, and A is the frequency factor.

**Figure 8:**
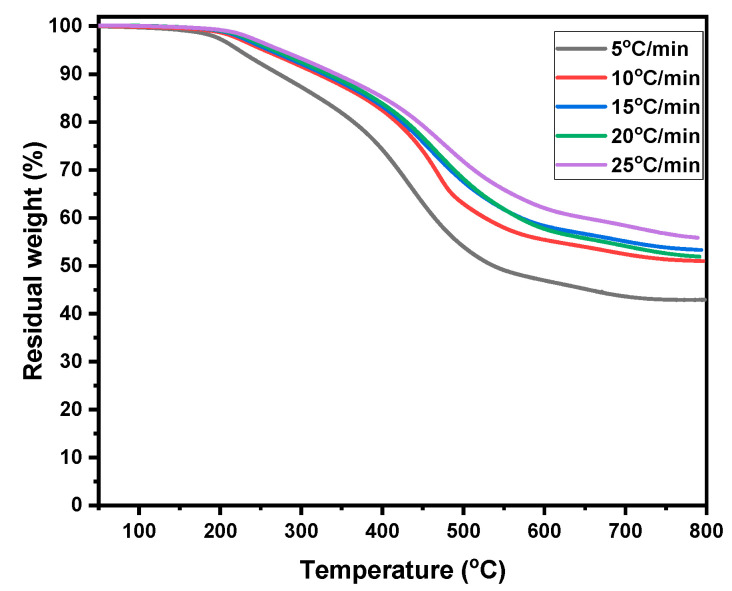
TGA thermograms of poly(CA-pdms-fu) at different heating rates.

A series of TGA thermograms of the poly(CA-pdms-fu) was measured at different heating rates of 5, 10, 15, 20, and 25 °C/min, as shown in Figure 8.

**Figure 9:**
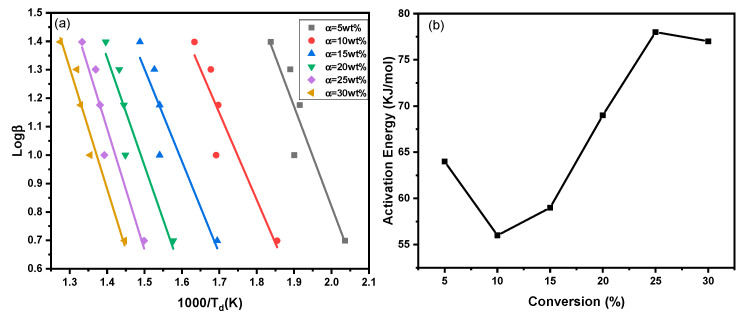
(**a**) Flynn-Wall-Ozawa isoconversion plot for calculation of activation energy of poly(CA-pdms-fu) and (**b**) Degradation activation energy as a function of the conversion of poly(CA-pdms-fu).

Figure 9a shows the plot of log_10_β versus 1000/*T*_d_. The conversions (α) used for each heating rate were 5%, 10%, 15%, 20%, 25%, and 30%. Figure 9b shows the activation energy for each conversion.

The activation energy increased from 56 kJ/mol, achieving a maximum value of 78 kJ/mol at 10 to 25% of conversion, respectively. This increase of E_a_ might be associated with the crosslinking reaction which usually demonstrates a change in E_a_.

To further characterize this elastomeric polybenzoxazine as an excellent flame-retardant material, the limiting oxygen index (LOI), which is the minimum oxygen concentration in an oxygen and nitrogen mixture, in percentage, that will support the continuous combustion of polymers, was estimated. This parameter can be calculated from the van Krevelen Equation (2) using the char yield values (CY) obtained from TGA analysis as follows:

LOI= 17.5 + 0.4 (CY)(2)

When the LOI value is ≤ 26, the material is considered flammable [63,64]. The LOI calculated for the poly(CA-pdms-fu) is 38, which can be considered at least a self-extinguishing polymer. This property is very attractive since this is a green polymer and does not make use of a halogenated component.

Microscale combustion calorimeter (MCC) has been used to measure the flammability of material through the heat release rate using milligram specimen mass. This method effectively eliminates the diffusion component from the flammability characteristics as the sample size is extremely small in the milligram scale. The hazardous fire information that MCC can provide includes specific heat release rate (SHRR) of the sample per unit weight (W/g) as a function of temperature, heat release capacity (HRC) known as η_c,_ and total heat release rate (THRR) [65]. Heat release capacity can be calculated using the maximum specific heat release rate according to Equation (3):

(3) $$\eta_{c=\frac{q_{\max}}{\beta }}$$

where q_max_ is the maximum value of SHRR (W/g), and β is the average heating rate over the measurement range, K.s^−1^. When the heating rate of 1 °C/s is used, the specific heat release rate per gram (W/g) has the same value as the HRC in J/g K when the degradation process happens in a single mechanism, as aforementioned in the activation energy of degradation section. Heat release capacity has become an important parameter to determine the material flammability and is considered as one of the most quantitative parameters for flammability determination [65,66,67,68].

**Figure 10:**
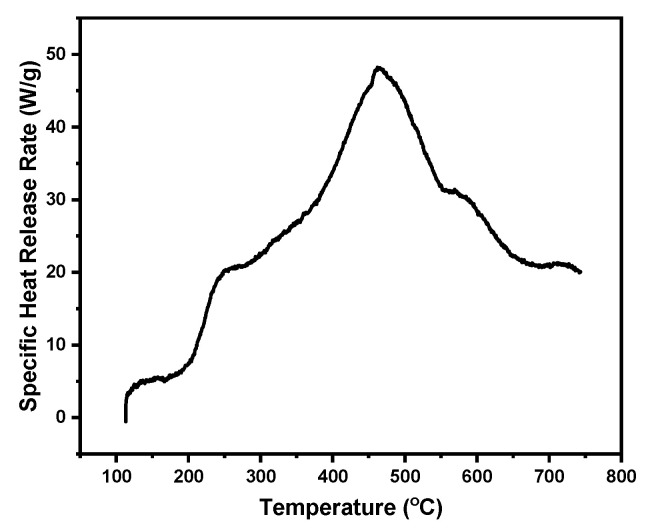
MCC curve of poly(CA-pdms-fu).

Figure 10 shows the specific heat release rate (and the heat release capacity since the heating rate used was 1 °C/s) poly(CA-pdms-fu) as a function of the temperature.

The poly(CA-pdms-fu) has a low heat release capacity of 48 J/g·K. We now have a flexible elastomer that has excellent flame resistance, lower than engineering polymers, such as polyetherimide, polyphenylsulfones, and polyether ketone, that present values around 100 J/g.K, based on the microscale combustion calorimeter results [69]. Furthermore, when the HRC of material is below 300 J/g.K, it is considered self-extinguishing material. Moreover, when it is below 100 J/g.K, it is non-ignitable [32]. After analyzing all characteristics related to fire resistance and decomposition, such as TGA, activation energy, LOI, and HRC, this elastomeric polybenzoxazine, which is free of halogenated flame retardant or phosphorous compounds and has natural renewable component, can be considered a self-extinguishing polymer.

As this elastomeric polymer presented extraordinary results as flame retardant polymer, a nanocomposite was prepared to be used as a very thin coating for polyurethane foam. The formation of nanocomposite depends on the compatibility of polymer and inorganic material, and not all combinations of polymer and inorganic additive will work as a good nanocomposite. This necessitates the actual trial to seek the optimal conditions, such as component concentration, interphase engineering, and processing conditions.

**Figure 11:**
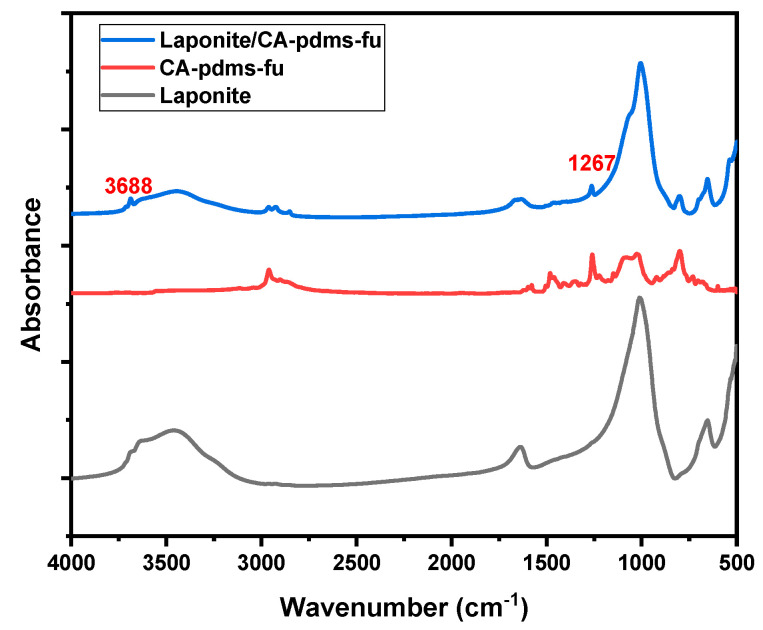
FTIR spectra of Laponite/CA-pdms-fu, CA-pdms-fu and laponite.

Fourier transform infrared spectroscopy (FT-IR) has been used to determine the chemical interaction and functional groups of the polymer/inorganic filler nanocomposite. Figure 11 shows the spectrum of poly(CA-pdms-fu) with its characteristics benzoxazine ring absorbance, as mentioned in the previous section. Moreover, the broad stretching vibration of OH groups in the 3443 cm^−1^ and 651 cm^−1^ bending vibration of the OH group, and the Si-O stretching modes occur at 1008 cm^−1^. There is a weak vibration at 3688 cm^−1^ of M_g_OH which is near free OH stretching mode of the hydroxyl groups confined in the silicate lattice of laponite [70,71]. The presence of CA-pdms-fu in Laponite/CA-pdms-fu influences the position of the absorption band of functional groups of laponite. The OH band at 3688 cm^−1^ of laponite becomes more visible after the addition of CA-pdms-fu. This is likely due to the reduction of the broad band around 3400 cm^−1^ which is in part due to the OH stretching mode of the adsorbed water on the laponite surface. Polybenzoxazine is much more hydrophobic and the good interaction between the laponite surface and the matrix discourages the presence of physically adsorbed water on the surface. This is a circumstantial evidence that the original attempt of mussel-mimetics to develop a polymer that adheres well to hydrophilic surface was successful. However, since the band at 3680 cm^−1^ is due to the hydroxyl groups that are hidden inside the silicate gallery, the presence of the polymer matrix will not visibly alter its intensity. Thus, this weak band becomes more visible in the nanocomposite spectrum. The weak but characteristic Si–CH_3_ band at 1267 cm^−1^ is clearly visible showing the presence of CA-pdms-fu.

**Figure 12:**
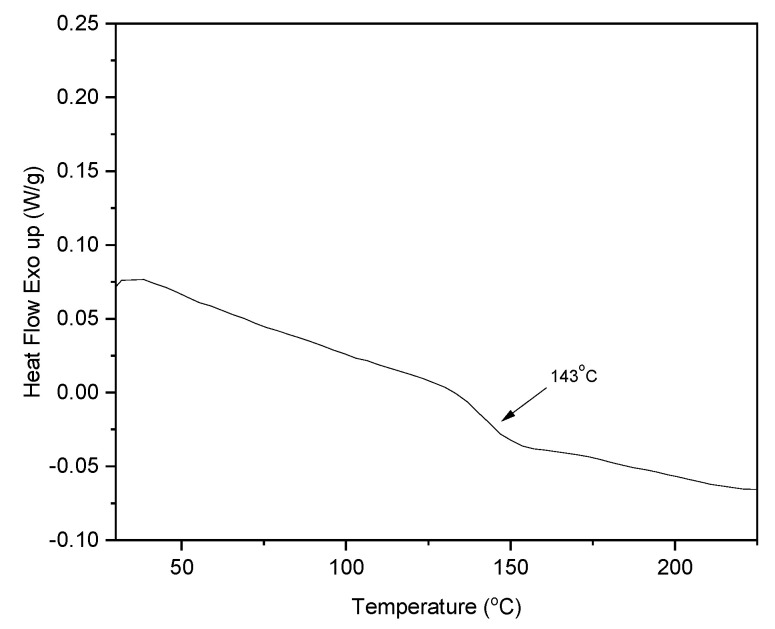
DSC thermogram of Laponite/poly(CA-pdms-fu).

DSC was also used to investigate the thermal behavior of the polymer-clay nanocomposite. The glass transition temperature (*T*_g_) of poly(CA-pdms-fu) nanocomposite shown in Figure 12 reveals that *T*_g_ increased from 73 °C for the unfilled polybenzoxazine to 143 °C for the nanocomposite. This increase could be due to the interaction of the very thin polymer with the surface of laponite fillers. Further detailed study is needed to evaluate the possibility of microphase separation as well as the interaction with the surface.

**Figure 13:**
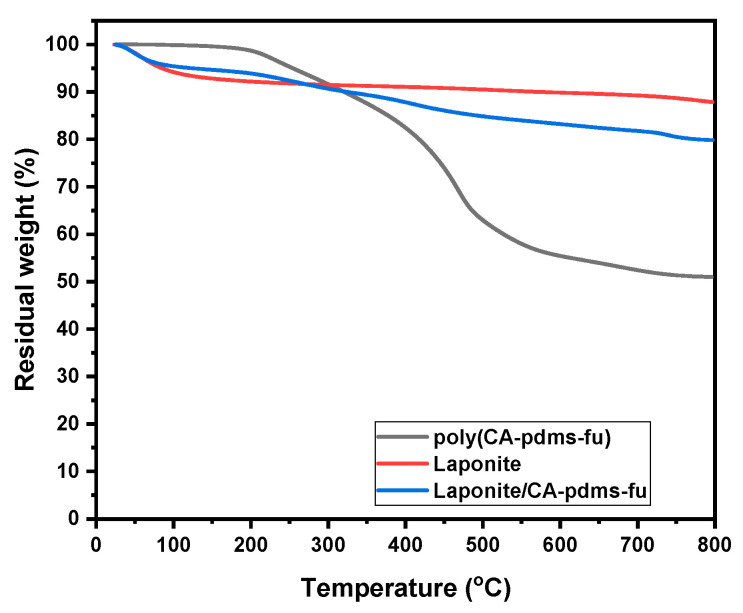
TGA thermograms of nanocomposite and neat polymer and laponite.

The TGA thermogram of the nanocomposite, shown in Figure 13, displays an expected increase in the char yield compared to the polymer itself due to the very high laponite content. Laponite is a synthetic clay with the coin-like shape of stacked silicate layers with diameter of approximately 25 nm. It has a unique charging state in water where the basal plane is negatively charged whereas the edge plane is positively charged [72]. Thus, it could form a T-shape interaction of two particles having the edge of one particle is interacting with the basal plane of another. This configuration is herein called T-bond. Another possible configuration is called parallel, partially overlapped (PPO) where all the particles are aligned in a single direction with part of the discs are overlapped [73]. Although it is not the main goal of the current paper to identify detailed nanoparticle alignment, as we attempt to achieve overall layered morphology of the nanocomposite, some would find it to be beneficial to learn detailed particle-particle interaction and/or alignment for further detailed control of the morphology. Using laponite as a reinforcing filler, a layer-by-layer technique was used to obtain a brick-and-mortar structure where laponite filler is tightly aligned in one direction with a comb-like molecule, poly(L-lysine)-g-poly(ethylene glycol), as a very small amount of adhesive. A very high nanofiller-content nanocomposite was obtained. The authors also followed a formation of a liquid crystalline texture as the suspension was being dried [74]. It is this very morphology that the current paper attempts to form using laponite and newly synthesized flame-retardant elastomeric polybenzoxazine.

**Figure 14:**
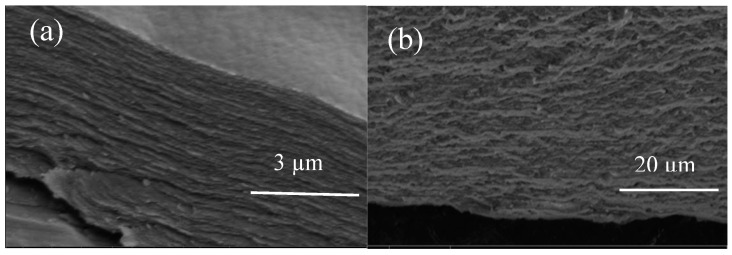
SEM images of (**a**) Laponite and (**b**) Laponite/ CA-pdms-fu.

As a result of the amount and structure of laponite in combination with the excellent char yield obtained from polybenzoxazine, it is assumed that an increase in thermal stability is due to the formation of char. The laponite:polymer ratio used is 90:10 wt%. However, the observed weight reduction from pure laponite is approximately 8% around 800 °C. It is likely that this reduction of weight loss, or increased char yield, is due to the synergism of nanocomposite formation beyond the expected char yield based on the composition. The layered morphology is acting as a diffusion barrier, thus increasing the residence time of molecular fragments forming char due to the secondary reactions. Formation of secondary products resembling base structure of a char has been observed during the GC-Mass analysis of degrading polybenzoxazine fragments that are kept in the same container [75,76]. The nanocomposite forms a tortuous pathway that limits the diffusion of volatile decomposed material that form during thermal degradation out of the nanocomposite. Similarly, the nanoplatelets neatly stack in a plane orientation that constrain the diffusion of gases inside the nanocomposite and restrict the contact of polybenzoxazine with the oxidizing environment, as shown in Figure 14 [77].

SEM photomicrographs of cross-sectional morphology of fractured laponite and nanocomposite films are shown in Figure 14. Morphology of laponite film depicts a very oriented nanoplatelets sheet structure. The nanocomposite containing 90 wt% laponite and 10 wt% CA-pdms-fu show a similar layer arrangement of the nanoplatelets, confirming the nacre-mimetic self-ordering of a brick-and-mortar structure of ultra-high nanofiller nanocomposite. However, there are few positions lacking a layered structure that reveal some protruding, suggesting that the nanocomposites are not perfectly homogeneous.

**Figure 15:**
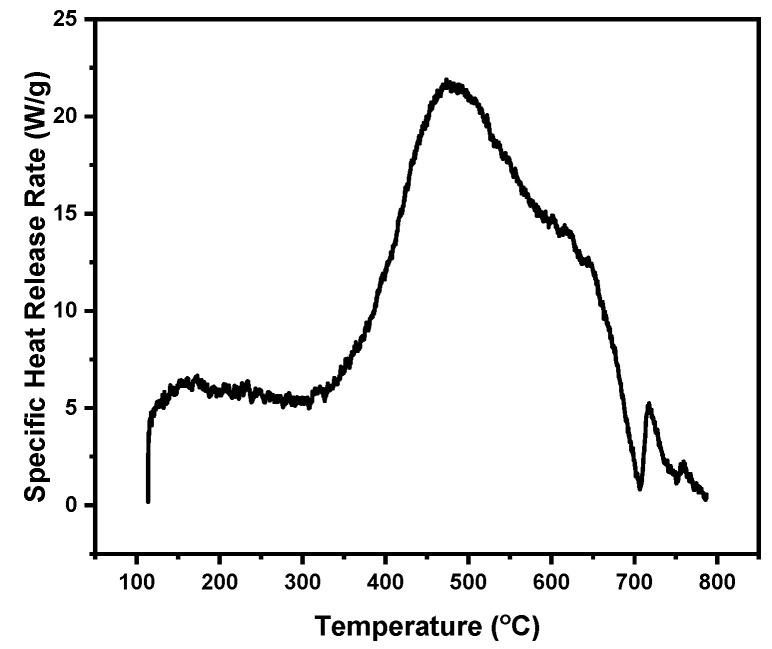
MCC curve of Laponite/CA-pdms-fu.

MCC was used to evaluate the flammability behavior of the nanocomposite, Laponite/ CA-pdms-fu. Figure 15 shows the specific heat release rate and the heat release capacity as a function of the temperature. As aforementioned, when the HRC of material is below 100 J/g.K, it is non-ignitable [32]. The nanocomposite developed presented non-combustible behavior by showing an extremely small heat release capacity of 23 J/g.K.

The characterization of the new flame retardation technology showed excellent properties to be used as a flame-retardant material. Based on the properties of this green flame-retardant nanocomposite, a very thin nanocomposite coating was applied to a polyurethane foam. Using a single dip-coating technique, polyurethane foams with different uptakes were obtained.

**Figure 16:**
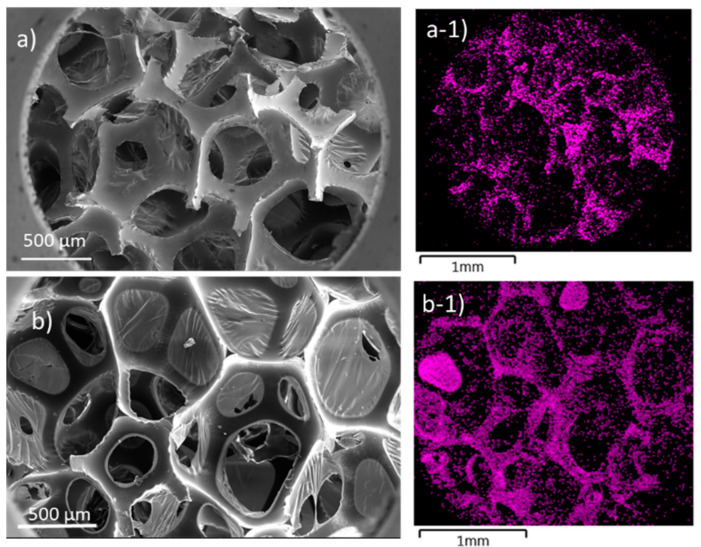
SEM imagines of coated PUF with 90/10 wt% of Laponite/CA-pdms-fu with different uptakes and Mapping of the Si element on coated PUF with 90/10 wt% of Laponite/CA-pdms-fu with different uptakes (**a**,**a-1**) 4 wt% of load, image from the center part of PUF and (**b**,**b-1**) 6 wt% of load, image from the upper edge of PUF.

EDX analysis was used to evaluate the even distribution of the coating on PUF, and the distribution of silicon was used as a target element due to its absence on PUF chemical structure. Coated PUF at different uptake (4 and 6 wt%) displayed a homogeneous dispersion of the film, as can be seen in Figure 16. The uniform distribution of the silicon on the PUF indicates that the coating procedure proposed in the current study is efficient. All the EDX images taken from the upper edge, the lower edge, and center part of coated PUF showed an even distribution of the silicon element.

**Figure 17:**
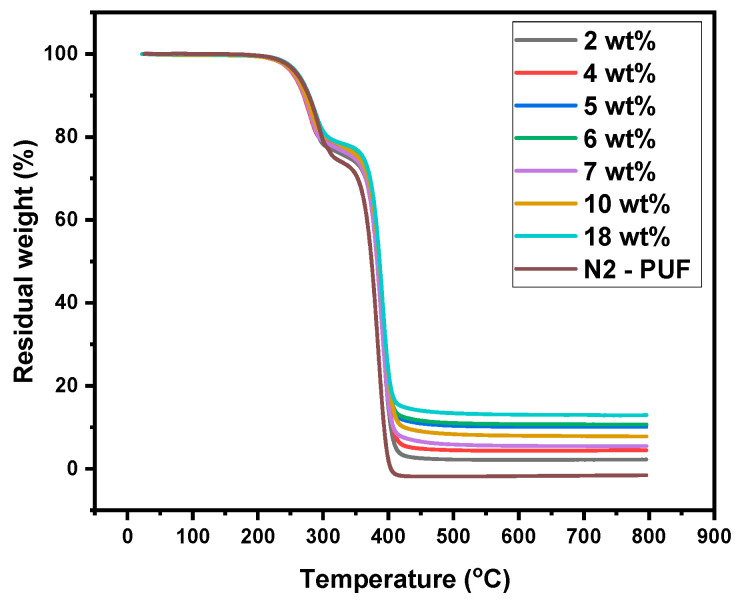
Laponite/CA-pdms-fu—90/10 wt% in different nanocomposite uptakes.

TGA was used to characterize the thermal degradation of PUF and coated PUF under nitrogen atmosphere. The thermogram of coated and uncoated PUF under the nitrogen atmosphere, which simulates the degradation mechanism in the condensate phase, is shown in Figure 17.

Figure 17 shows the nanocomposite coating in different loads on PUF. The PUF decomposition occurs in two stages. The first stage is the breakage of the urethane bond that happens between 212–332 °C, and the second stage that occurs above 300 °C is referred to as the volatilization of regenerated polyols. No relevant difference can be seen in terms of onset degradation temperature. However, there was a delay at the weight loss over the second stage. This indicates that the Laponite/CA-pdms-fu coating improves the char formation, which contributed to the delay of the second stage. The char formation increased for all the coated PUF due to the nanocomposite effect that decreases the amount of combustible gases released during the thermal decomposition of the material (Table 2).

**Figure 18:**
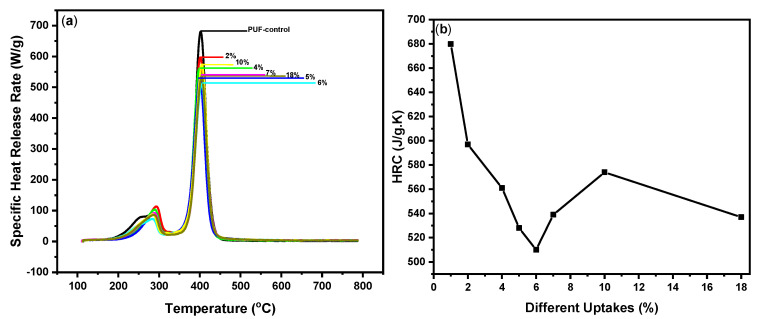
(**a**) MCC curve of coated PUF in different uptake, (**b**) Heat release capacity (HRC) of coated PUF in different uptake.

Microscale combustion calorimetry (MCC) was used to predict the flammability behavior of coated polyurethane foam. PUF with different uptakes of 2, 4, 5, 6, 7, 10, and 18 wt% was obtained. Figure 18a,b shows the plots of specific heat release rate and the heat release capacity as a function of the temperature. The heat release capacity (HRC) and char yield of each sample are presented in Table 3. At the up-take of 4%, the char yield suddenly increased. This might be related to the minimum thickness requirement to maintain sufficient residence time of the fragmented molecules to undergo secondary reactions to form chars. It appears that the transition is in the vicinity of 3% uptake.

There are two peaks observed in the MCC tests for coated and uncoated PUF. The first peak is attributed to the combustion of the degradation of a diisocyanate component, and the second peak is the decomposition of a polyol component of the polyurethane foam [78,79]. These results are in agreement with TGA results.

The HRC of all coated PUF samples decreased when compared with the uncoated polyurethane. The coated PUF in 6% uptake presented the best result which exhibits the reduction by 25%, as shown in Table 3 and Figure 18a,b. The result might be associated with the combination of laponite, and polybenzoxazine that increases the char formation from 9.8% for the uncoated PUF to 84% for the coated PUF in 6% uptake. It is an impressive result because, in this work, the best result to HRC used just 6% in loading to coat PUF from a 1wt% solution, while other articles used 20% loading to achieve 30% increase in char yield, although it is for cotton fabric [80]. Unfortunately, no direct comparison can be made since we could not find a paper that used MCC for coated PU foam. This high char formation would eliminate the dripping of molten PUF during combustion. The char yield suppresses the oxygen diffusion, which prevents combustible molecular fragments from further reacting with the oxygen and generating heat. This indicates that the very thin coating on the polyurethane foam (PUF) obtained through a simple, single dip-coating with the nacre-mimetic self-ordering of a brick-and-mortar structure is effective as a green flame-retardant barrier.

**Figure 19:**
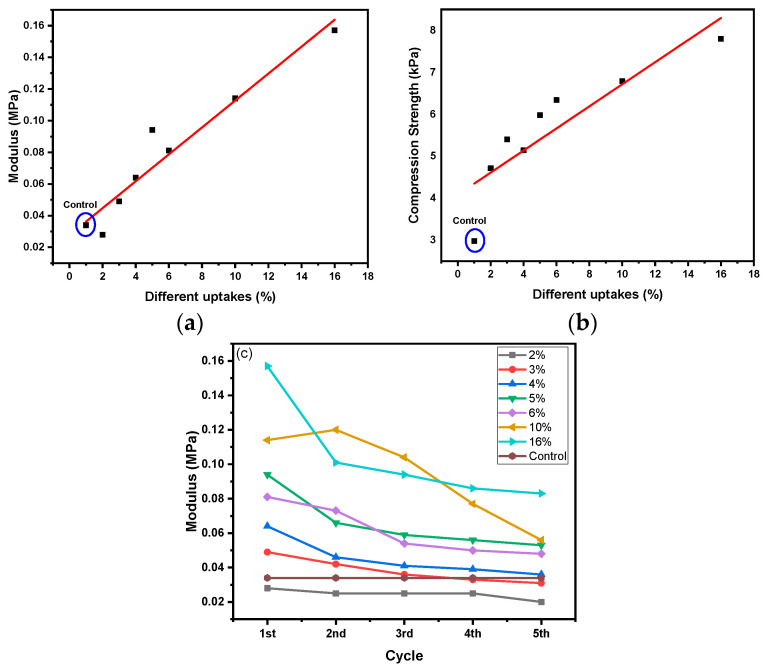
Result of compression test for uncoated and coated PUF in different uptakes. (**a**) Modulus results, (**b**) Compression strength, and (**c**) repeated modulus test.

PU foam is often used as a material for furniture. As such, the mechanical feel is an important requirement. Therefore, coated PUF at different loads was produced to understand the limit of coating up-take that should be applied on the foam without increasing the stiffness substantially. A compression test was used to evaluate the stiffness, as shown in Figure 19. Each coated PUF was submitted to the compression test for five consecutive times, in order to simulate how foam would be used in real life. It is known that modulus and compression strength is dependent on the thickness of the coating. This statement is in agreement with the results found in this work, as can be seen in Figure 19a,b. In general, the modulus and compression strength increased with increasing the coating uptake on PUF. Figure 19c shows that the greater the uptake of nanocoating, the higher the modulus. However, after repeated modulus tests on the same sample, the modulus decreased relatively quickly. The coated PUF with 3% and 4% of uptake revealed the best performance since the fifth repeated modulus test achieves the same value as the uncoated sample. However, the coated PUF with 5%, 6%, and 7% also presented promising results. These samples appear to be the best choice to proceed with the flammability test.

**Figure 20:**
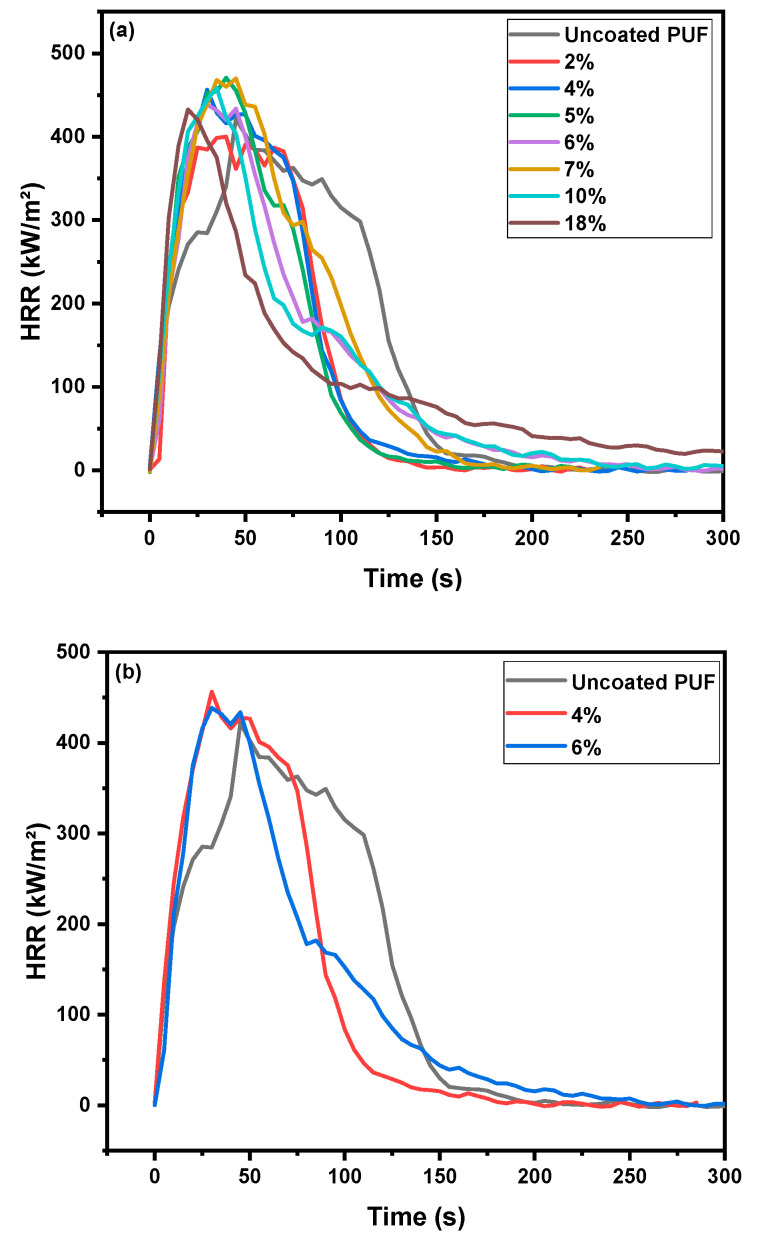
(**a**) and (**b**) Heat release rate plotted versus time for uncoated and coated PUF.

Cone calorimeter was used to assess the fire performance of the uncoated and coated polyurethane foam by measuring peak heat release rate (pHRR), total hear release (THR), total smoke rate (TSR), and char residue. Figure 20a shows the pHRR test at 35 kWm^−^^2^ for uncoated and coated PUF in different mass gain and Figure 20b shows the outstanding results of coated PUF with low mass gains. The first peak HRR of uncoated PUF indicates its combustion, which causes the collapse of the foam, which occurs at 285 kWm^−^^2^ at 25 s. The second pHRR exists at 483 kWm^−^^2^ at 45 s. This second peak refers to pool fire [78,81]. These two peaks correspond to the combustion of isocyanate and polyol of polyurethane foam [82]. The cone calorimeter data is summarized in Table 4.

**Figure 21:**
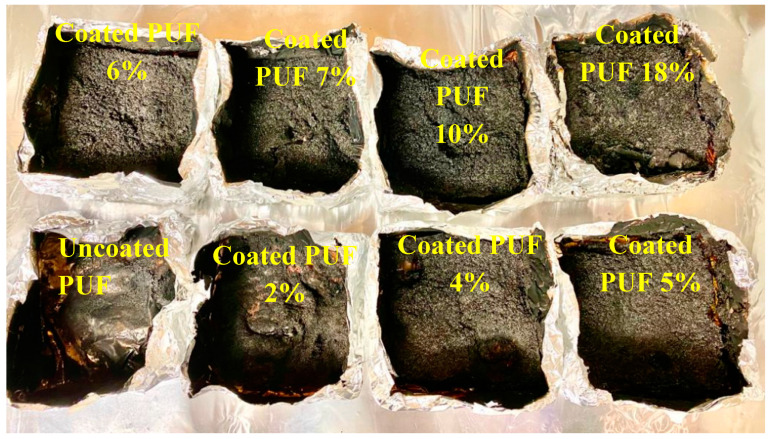
Optical photographs of uncoated and coated polyurethane foam with different uptake after cone calorimeter test.

Coated polyurethane foam shows a higher development of the first peak of HRR than uncoated foam, due to the polymer portion in the nacre-mimetic brick-and-mortar structure of the nanocomposite. In addition, the high clay content might increase the HRR by the existence of catalytic sites on laponite. This catalyst site initially promotes the combustion until a protective char layer is formed. This increase in the first pHRR was also observed by Davis and collaborators that used a polymer composite based on organoclay [11]. However, the second pHRR decreases for all the coated foam. For samples with 4% and 18% gain mass, a second peak was not observed. For both cases, it indicates that pool fire was not formed, which can decrease the fire threat significantly. The coating alters the PUF’s burning behavior, eliminating the melt dripping by creating a carbonaceous protective layer that maintains the initial shape of PUF [83], as seen in Figure 21. Thus, the combustion rate of combustible fragments decreases, reducing the fire source that further burns the material. The melted material is an igniter that increases the flame propagation in soft furnishings by 35%. The influence of the melted pool as a fire threat is not predicted in a standard cone calorimeter. Consequently, it is possible to add a 35% reduction in pHRR if it measured in a full-scale test [11,84].

The char yield for almost all the samples increased with the mass gain. Howbeit for 7% mass gain the char yield decreased. The phenomenon might be related to the evaporation of the material, which lets less material behind decreasing the char formation.

The total smoke release of the coated sample had a considerable reduction compared to uncoated polyurethane foam when the mass gain is high. TSR is an essential parameter since materials can produce toxic smoke and gases during combustion. Inhalation of toxic gases is one of the main reason of death related to fire events [85].

Table 4 shows the total heat release (THR) of uncoated and coated polyurethane foam. By comparing those values, it is evident that the very thin coating is suppressing the total amount of heat generated, acting as an insulating barrier, which decreases the amount of available combustible species.

## 4. Conclusions

The single dip-coating approach appears to be a fast and effective method used to prepare the ultra-thin coating on the polyurethane foam (PUF) as a green flame-retardant barrier. The nanocomposite fabricated from Laponite/CA-pdms-fu presented the desired brick-and-mortar structure, which significantly assists in reducing the PUF flammability. The green flame-retardant nanocomposite decreased by 62%, the second pHRR of the PUF, using just 6% mass gain. Emphasizing that this approach consists of single dip-coating, using a very low concentration of nanocomposite solution, the proposed method is a readily scalable to products of any dimensions. The modulus of coated PUF foam with 6% of uptake showed an acceptable stiffness since the fifth repeated modulus test starts approaching the values of the control PUF.

## Figures and Tables

**Table 1:** Tensile Properties of polybenzoxazine containing PDMS.

Code	Elastic Modulus (MPa)	Tensile Strength (MPa)	Elongation at Break (%)	Approach for Toughness
Poly (BA-a) [47]	3300	35	1.6	-
**Poly (CA-pdms-fu)**	**65**	**4**	**25**	**Copolymerization**
Poly (BA-a)/PDMS (850) [47] *	330	9	3.8	Copolymerization
Poly (BA-a)/PDMS (1622) [47] **	20	0.04	7.1	Copolymerization
Poly (BA-a)/PDMS (7%) [58] †	2600	42	3.5	Hybridization
Poly (BA-a)/PDMS (13%) [58] †	2200	48	4.4	Hybridization
Poly (BA-a)/PDMS (5%) [60] ‡	2800	38	2.6	Hybridization
Poly (BA-a)/PDMS (10%) [60] ‡	2600	42	3.5	Hybridization
Poly (BA-a)/PDMS (20%) [60] ‡	2200	48	4.4	Hybridization
Poly (BA-a)/PDMS (30%) [60] ‡	Macroscopic phase separation			Hybridization
Poly (BA-a)/PDMS (20%) [59] ⁑	2200	36	4.3	Hybridization
Poly (BA-a)/PDMS (30%) [59] ⁑	2200	56	4.6	Hybridization
Poly (BA-a)/PDMS (40%) [59] ⁑	2200	56	4.4	Hybridization

* Molecular weight = 850 g/mol and reference; ** Molecular Weight = 1622 g/mol and reference; † Molecular weight = 550 g/mol and reference; ‡ molecular weight = 700 g/mol and reference; ⁑ Molecular weight = 460–780 g/mol and reference.

**Table 2:** Char yield percentage of the coated PUF for different uptake.

Nanocomposite/Uptake	Char Yield (%)
Polyurethane Foam	0.36
Laponite/CA-PDMS-fu—2 wt%	2.26
Laponite/CA-PDMS-fu—4 wt%	4.49
Laponite/CA-PDMS-fu—5 wt%	10
Laponite/CA-PDMS-fu—6 wt%	10.73
Laponite/CA-PDMS-fu—7 wt%	5.51
Laponite/CA-PDMS-fu—10 wt%	7.8
Laponite/CA-PDMS-fu—18 wt%	12.97

**Table 3:** Heat release capacity and char yield percentage of the coated PUF for different uptakes.

Sample	Equation (3)	First Peak Max. T(°C)	Equation (3)	Second Peak Max. T(°C)	Char Yield (%)
	1st Peak HRC (J/g.K)		2nd Peak HRC (J/g.K)		
**Control PUF**	**85**	**287**	**680**	**403**	**9.8**
Coated PUF 2%	113	294	597	400	10.2
Coated PUF 4%	103	292	561	398	76
Coated PUF 5%	89	289	528	401	82
**Coated PUF 6%**	**71**	**287**	**510**	**406**	**84**
Coated PUF 7%	92	289	539	405	83
Coated PUF 10%	86	287	574	405	88
Coated PUF 18%	86	288	537	404	88

**Table 4:** Cone calorimetry results of uncoated and coated PUF.

Sample	1st PHRR (kW/m^2^)	2nd PHRR (kW/m^2^)	TSR (m^2^/m^2^)	THR (MJ/m^2^)	Char Residue (%)
**Uncoated PUF**	**285**	**483**	**227**	**41**	**1**
Coated PUF 2%	307	400	215	31	7
Coated PUF 4%	456	-	235	34	9
Coated PUF 5%	470	318	215	32	12
**Coated PUF 6%**	**438**	**182**	**215**	**34**	**15**
Coated PUF 7%	468	298	272	38	12
Coated PUF 10%	458	172	196	34	15
Coated PUF 18%	432	-	173	34	23
